# Trends in haemoglobin levels from 1968 to 2017 and association with hormonal contraceptives: observations from the population study of women in Gothenburg, Sweden

**DOI:** 10.1080/02813432.2023.2222767

**Published:** 2023-06-24

**Authors:** Linda Sandin, Amanda von Below, Maria Waller, Cecilia Björkelund, Ann Blomstrand, Rebecca Runevad, Dominique Hange

**Affiliations:** a Primary Health Care, School of Public Health and Community Medicine, Institute of Medicine, Sahlgrenska Academy, University of Gothenburg, Sweden; b Research, Education, Development & Innovation, Primary Health Care, Region Västra Götaland, Sweden

**Keywords:** Haemoglobin, anaemia, hormonal contraceptives, population study, women

## Abstract

**Aim:**

To investigate trends in the haemoglobin (Hb) level in middle-aged Swedish women from 1968 to 2017 and to examine the potential association between Hb and the use of hormonal contraceptives (HCs).

**Design:**

A prospective observational population study of representative 38- and 50-year-old women of Gothenburg, Sweden.

**Setting:**

The population study of women in Gothenburg started in 1968–1969 and has continued since then with new examinations every 12 years, including both follow-ups and new recruited cohorts. The study consists of both physical examinations and questionnaires.

**Subjects:**

Two thousand four hundred eighty-eight women aged 38 and 50 participated in the study from 1968 to 2017.

**Statistical methods:**

Linear regression model analyses were used to analyse linear and non-linear trends in the level of Hb. Linear and logistic regression models were used to analyse possible associations between HC and Hb and possible associations between the use of HC and anaemia, respectively.

**Main outcome measures and covariates:**

Hb was measured in g/L. HC included any ongoing use of HC therapy. Covariates were smoking, body mass index (BMI), alcohol consumption and education.

**Results:**

A non-linear U-shaped trend in mean Hb was seen in the two age groups, 38- and 50-years old. After adjusting for covariates, a significantly higher mean Hb was seen in the 2016–2017 examination compared to 1980–1981, 1992–1993 and 2004–2005. In 38-year-olds, using HC was associated with a reduced risk of anaemia (OR 0.35, 95% CI 0.13–0.75). In both age groups, the use of HC was significantly associated with having a higher Hb.

**Conclusions:**

Mean levels of Hb in middle-aged women of the general population seem to be increasing again after lower levels in the 1980s and 1990s. The use of HC was associated with having a higher Hb and a lower risk of anaemia in 38-year-old women.

## Key points

Studies of trends in haemoglobin (Hb) in Swedish women are scarce, although many factors associated with Hb levels have changed during the last decades.

The use of hormonal contraceptives was associated with higher Hb and decreased risk of anaemia in younger middle-aged women.

This study shows there is a non-linear (U-formed) trend in mean Hb, with increasing values since 1992.

## Introduction

Haemoglobin (Hb) can impact health as well as quality of life. Both anaemia and elevated levels of Hb have been shown to be linked to disease and health risks [1–5], and a significant association between the risk of stroke and Hb concentration has been observed also in previously healthy premenopausal women [6]. Low and high Hb levels have also been associated with increased all-cause mortality, while reaching and maintaining normal Hb levels have been shown to correlate with subsequent decreased mortality [6,7]. However, although Hb is an important marker of health, trends in Swedish women are relatively unexplored. Also, when a worldwide analysis was conducted, representative data on Hb levels were most sparse in high-income regions [8].

Globally, by far the largest health problem when it comes to deviant Hb levels is anaemia, defined as a reduced or insufficient number of erythrocytes to meet the body’s physiologic needs [9,10]. Anaemia is estimated to affect one quarter of the world’s population with highest prevalence found in preschool children and women [11]. The precipitating factors can be multiple, but the most common cause of anaemia is thought to be iron deficiency [9].

For women in developed countries, heavy menstrual bleeding is a well-known cause of iron-deficiency anaemia [12], and studies have suggested that hormonal contraceptives (HCs) can reduce menstrual bleedings [13]. Current users of HC were shown to have a higher Hb compared to non-users in a study including countries from Africa, the Americas and Asia [14]. In the same study, intra-individual increases in Hb were seen in women who initiated treatment with HC [14]. This was also true for women using levonorgestrel intrauterine devices in a randomised trial, where a statistically significant difference in Hb also was shown compared to users of a copper-releasing device [15]. Apart from HC, several other factors have shown to be associated with Hb levels. Higher Hb levels are seen in 40- and 50-year-old cigarette smoking women compared to non-smoking women of the same age [16]. A higher body mass index (BMI), as well as high socioeconomic status, has also been associated with a higher Hb in women [17], although not confirmed in all studies [16].

In Swedish women, several of the above factors have changed during the last decades. Smoking has decreased [18] and various studies show an increasing level of BMI [19,20]. Further, a previous study from the population study of women in Gothenburg has shown changes in the use of HC among both 38- and 50-year-old women, with a multiple increase in the use of intrauterine devices with levonorgestrel [21]. However, although changes have occurred in these associated factors, few studies have investigated the trends in Hb levels for Swedish women during the same time. Furthermore, to our knowledge, there are no studies examining whether Hb levels and the prevalence of anaemia are associated with use of HC in the general population of Swedish women. Most studies in the field concern women in low- and middle-income countries [22].

### Aim

The aim of this study was to investigate trends in Hb levels in Swedish 38- and 50-year-old women between 1968–1969 and 2016–2017. A second aim was to investigate whether there is an association between Hb level, prevalence of anaemia and/or high Hb, and the use of HC in the general population of middle-aged Swedish women.

## Materials and methods

### The population study of women in Gothenburg

The population study of women in Gothenburg is a prospective observational study initiated in 1968. Based on birth date (women born on the 6th, 12th, 18th, 24th and 30th day of each month), a representative sample of a total of 1622 women aged 38, 46, 50, 54 and 60 years were invited for a free health examination, including inquiry about health and social situation, medical examination and blood samples. Information was obtained from the Revenue Office Register. Of these, 1462 (90%) participated. Examinations including both follow-ups and new recruited cohorts of 38- and 50-year-old women have been conducted in 1980–1981, 1992–1993, 2004–2005 and 2016–2017. Participation rates have been 58–91%. A more detailed description of the study and characteristics of non-participants have been published elsewhere [18,21,23,24]. In total, 2488 women aged 38 and 50 years have participated between 1968 and 2017 (Table 1). In the present study, they were all included (1033 thirty-eight-year-olds and 1455 fifty-year-olds); a total of 603 (24.0%) women participated both as 38-year-olds and 50-year-olds.

**Table 1.** Participation rate in the population study of women in Gothenburg.

Examination year	38-year-olds, *n* (%)	50-year-olds, *n* (%)	Total, *n*
1968–1969	372 (91)	398 (91)	770
1980–1981	122 (85)	355 (82)	477
1992–1993	69 (72)	99 (76)	168
2004–2005	207 (60)	293 (58)	500
2016–2017	263 (63)	310 (73)	573
Total	1033	1455	2488

### Haemoglobin

Hb was measured in whole blood and is expressed in grams per litre (g/L). The level of Hb has been divided into subgroups where <120 g/L, 120–150 g/L and >150 g/L are considered as low, normal and elevated level of Hb, respectively. The upper limit was based partly on previous studies on high Hb and partly set to be close to the reference range for adult women (valid since 1 June 2004) according to Clinical Chemistry at Sahlgrenska University Hospital, which is specified to 117–153 g/L [25]. The Hb level used to diagnose anaemia was considered as <120 g/L, which is the limit used by the World Health Organization [9]. It can be difficult to classify anaemia in general practice [26].

Information regarding Hb level was available in 2469 of 2488 participants. Nineteen values were missing. The reasons for missing values were clotted blood sample (*n* = 2) and no blood sample due to fear of needles (*n* = 3) or due to difficult cannulation (*n* = 1). In 13 women, blood samples were missing due to unspecified reasons. One woman was excluded from the study because of greatly elevated Hb level due to an underlying medical condition.

### Hormonal contraceptives

Women who reported an ongoing contraceptive therapy containing hormones were included in the group HCs. HCs include contraceptive pills, hormonal intrauterine devices, contraceptive patches, implants, injections and vaginal rings. Information about the contraceptives was obtained from questionnaires answered by the women and from reported use of medications.

The participants who reported previous hysterectomy were excluded from the analyses regarding the effects of HC on Hb. This exclusion criterion was set since women who have had a hysterectomy neither have a menstrual blood loss nor are expected to use HC.

### Covariates

Covariates were measured at age 38 and 50 independently at every examination. Information about smoking, alcohol and education was obtained from the Medical Examination Questionnaire. Information about BMI was obtained from the registered weight and height at the examination.

#### Smoking

The participants were asked about their smoking habits and grouped into ‘non-current smokers’ (answers 0–3) and ‘current smokers’ (4–5), based on if they stated any type of current smoking in the questionnaire. The answer options were (0) non-smoker, (1) former smoker but not the last 15 years, (2) former smoker but not the last year, (3) stopped smoking during the last year, (4) still smoking (cigarettes) but do not inhale and (5) still smoking (cigarettes) and inhale.

#### Body mass index

BMI was calculated as body weight (in kg) divided by body height (in m^2^).

#### Alcohol

The participants were asked about the frequency of intake of beer, wine and spirits.

The answer alternatives were (1) never, (2) earlier, but not in 10 years, (3) earlier, but not in the last 12 months, (4) some days per month, (5) weekly, (6) several days per week and (7) daily. The answers were grouped into ‘never’ (never to not in the last 12 months), ‘sometimes’ (some days per months to weekly) and ‘regularly’ (several days per week or more) and coded as 0, 1 and 2.

#### Education

The educational level was categorised into three different groups. Primary school, secondary school and upper secondary school or university were defined as low, intermediate and high degree of education, respectively. In the examination 1968–1969 and 1980–1981, primary school (equal to or less than seven years), secondary school (equal to or less than nine years) and upper secondary school (more than nine years) or university were defined as low, intermediate and high degree of education, respectively. In the examination 2004–2005 and 2016–2017, the corresponding categorisation was primary school (equal to or less than nine years), secondary school (9–12 years) and university. Information regarding educational level from the examination 1992–1993 was missing.

### Statistics

Mean value and standard deviation (SD) were used for descriptive statistics. Differences in mean values between groups were analysed with analysis of variance (ANOVA) and post hoc test. Univariate ANOVA was performed to analyse trends in Hb and chosen covariates, with the latest examination (2016–2017) used as the reference group. Linear regression model analyses were performed to test cohort trends, and differences in trends in linear and non-linear trends in level of Hb. Logistic regression analyses were used to analyse linear and non-linear trends in prevalence of anaemia and elevated Hb. Linear and logistic regression models were also used to analyse possible associations between HC and Hb and between use of HC and anaemia, respectively. Statistical significance was accepted at *p* < .05. Software used was SPSS for Windows version 27 (SPSS Inc., Chicago, IL).

## Results

### Trends in Hb

**Figure 1.**
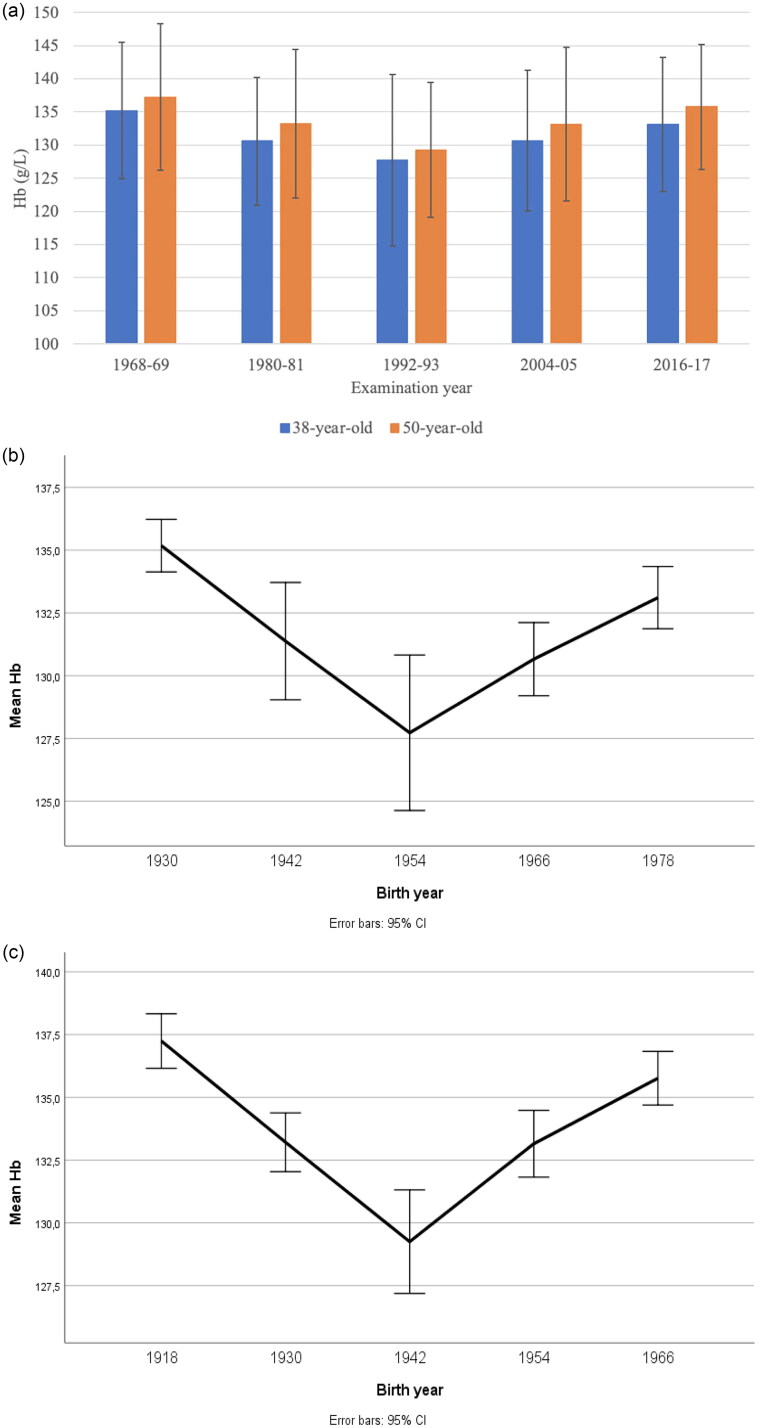
(a) Level of Hb in different examination years, mean values and SD. (b) Mean Hb level (g/L) in 38-year-old women. (c) Mean Hb level (g/L) in 50-year-old women.

The mean values of Hb in 38- and 50-year-old women during different examination years are shown in Figure 1. A significant non-linear U-shaped trend, with highest values in the first and the last examination, and lowest in the examination 1992–1993, was found for both age groups (*p* < .001). Lowest mean values were seen in 1992–1993 (127.8 and 129.2 g/L for 38- and 50-year-olds). In later examinations, Hb increased and reached almost as high in 2016 (133.1 and 135.7) as in 1968 (135.2 and 137.2). For all numeric mean values and SDs, see Appendix Table A.

Differences in Hb between different examination years were further analysed. Tables 2a and 2b show the individual differences between all examination years. Significant changes in the mean values were mainly seen when comparing the first examination (1968–1969) to the following three, whereas there was no significant change between the first and the last examination (2016–2017).

**Table 2a.** Differences in mean values in Hb between 38-year-olds in different examination years.

Examination year	1980–1981	1992–1993	2004–2005	2016–2017
1968–1969	4.6 (1.1)***	7.5 (1.4)***	4.5 (0.9)***	2.1 (0.8)
1980–1981		2.9 (1.6)	–0.1 (1.2)	–2.5 (1.1)
1992–1993			–2.9 (1.5)	–5.4 (1.4)**
2004–2005				–2.5 (1.0)

Numbers describe changes in mean values in Hb (g/L) between different examination years, standard error in parentheses. A negative value indicates the mean value for the examination year in the column is greater than the mean value in the row.

*p < .05.

** *p* < .01.

*** *p* < .001.

**Table 2b.** Differences in mean values in Hb between 50-year-olds in different examination years.

Examination year	1980–1981	1992–1993	2004–2005	2016–2017
1968–1969	4.0 (0.8)***	8.0 (1.2)***	4.1 (0.8)***	1.5 (0.8)
1980–1981		4.0 (1.2)*	0.06 (0.9)	–2.6 (0.8)*
1992–1993			–3.9 (1.3)*	–6.5 (1.3)***
2004–2005				–2.6 (0.9)*

Numbers describe changes in mean values in Hb (g/L) between different examination years, standard error in parentheses. A negative value indicates the mean value for the examination year in the column is greater than the mean value in the row.

* *p* < .05.

*** *p* < .001.

When a univariate ANOVA was performed with adjustment for covariates, significant changes in Hb were shown between the reference group 2016–2017 and the examination years 1980–1981, 1992–1993 and 2004–2005 (Table 3).

**Table 3.** Changes in Hb after adjustments for covariates, with examination 2016–2017 used as a reference.

Examination year	38-year-olds	50-year-olds
	*B*-coefficient (95% CI)	*B*-coefficient (95% CI)
2016–2017	0	0
2004–2005	–2.7 (–4.7 to −0.8)**	–2.3 (–4.0 to −0.6)**
1992–1993	–5.7 (–8.7 to −2.8)***	–6.6 (–9.2 to −4.1)***
1980–1981	–3.4 (–6.2 to −0.6)*	–2.8 (–4.8 to −0.8)**
1968–1969	1.7 (–0.7 to 4.2)	1.5 (–0.6 to 3.5)
Covariates		
Hormonal contraceptives	2.4 (0.6–4.2)**	3.0 (0.8–5.2)**
BMI	0.4 (0.2–0.6)***	0.3 (0.2–0.5)***
Smoking	3.4 (1.8–5.1)***	4.7 (3.5–6.0)***
Alcohol		
Wine	0.4 (–0.6 to 1.4)	1.0 (0.1–1.8)*
Beer	0.1 (–0.8 to 1.0)	–0.04 (–0.8 to 0.7)
Spirits	0.2 (–1.2 to 1.5)	–0.8 (–1.8 to 0.3)
Education	0.2 (–1.0 to 1.4)	–0.2 (–1.1 to 0.8)

Dependent variable: Hb. *B*-coefficient: the degree of change in the dependent variable for every unit of change of the independent variable.

* *p* < .05.

** *p* < .01.

*** *p* < .001.

#### Hb and covariates

Significant associations were found between Hb and HC, BMI and smoking, respectively, in all women. Regarding the association between alcohol consumption and Hb, a weaker association was found for consumption of wine in 50-year-old women, whereas no associations were seen for other types of alcohol or ages (Table 3). HC was further analysed separately.

#### Anaemia and elevated Hb

**Figure 2.**
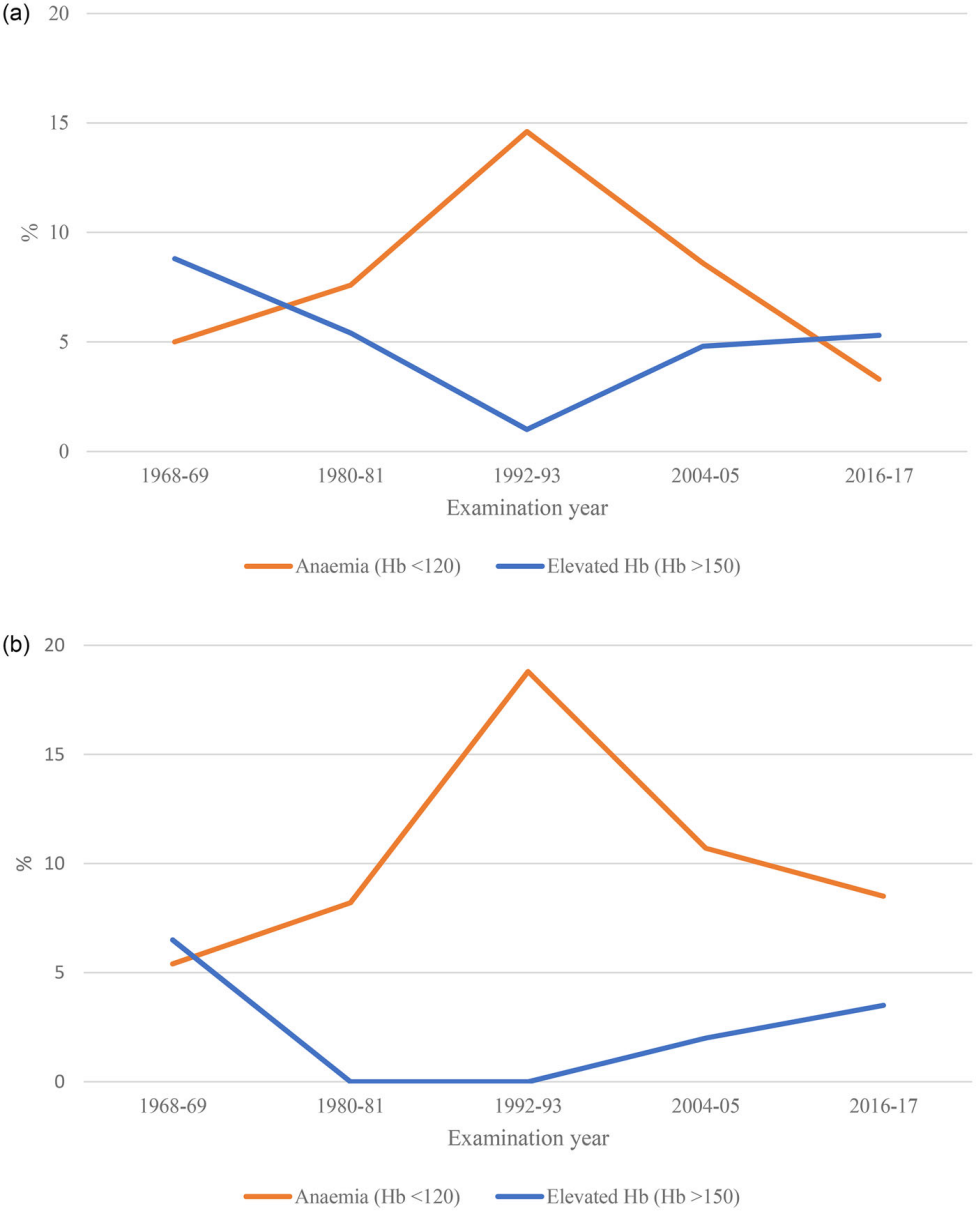
(a) Trends of anaemia and elevated Hb in 38-year-olds. Results from logistic regression models show significant non-linear trends in anaemia and elevated Hb, *p* = .004 and .0002, respectively. (b) Trends of anaemia and elevated Hb in 50-year-olds. Results from logistic regression models show significant non-linear trends in anaemia and elevated Hb, *p* = .0004 and .01, respectively.

Significant non-linear (U-formed and inverted U-formed) trends were seen in both 38- and 50-year-old women, regarding both anaemia and elevated Hb. A prominent peak in the prevalence of anaemia occurred in 1992–1993, which was also one of the years where almost none of the women had an elevated Hb (Figure 2(a,b)). For most of the examination years in this study, anaemia was more common than having an elevated Hb. However, in the last examination, the prevalence of Hb and the prevalence of anaemia approached one another again, and there were no longer any large differences in the prevalence of anaemia and elevated Hb in either 38- or 50-year-old women.

### Hormonal contraceptives and Hb

**Figure 3.**
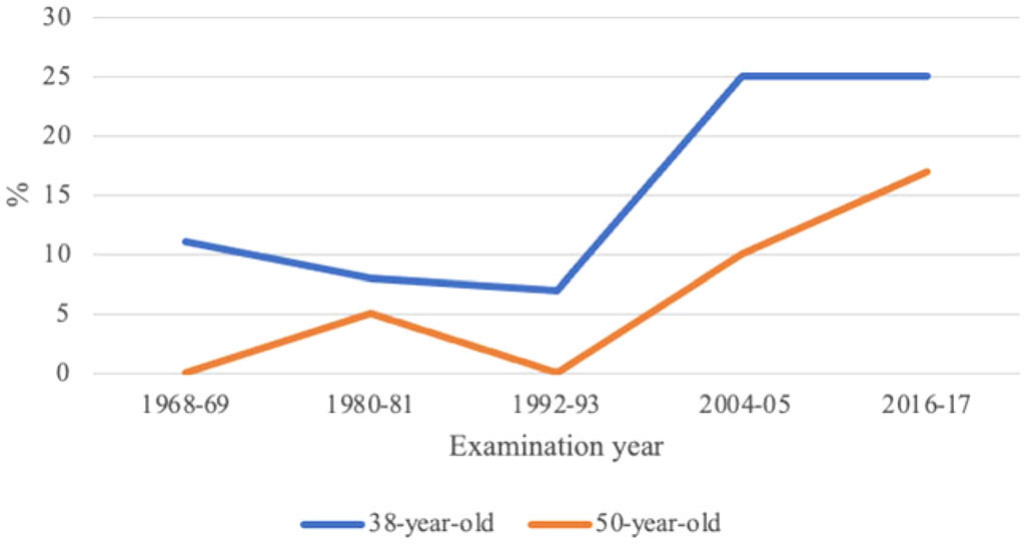
Use of hormonal contraceptives.

Figure 3 shows use of HC in different examination years, with increasing use in both age groups from the examination 1992–1993 and forward.

Using HC was significantly associated with having a higher Hb, both in 38- and 50-year-old women (*B* = 2.1, *p* = .02 and *B* = 2.7, *p* = .02, respectively). When adjusting for the covariates birth year, BMI, smoking, education and alcohol use, the association between Hb and HC was even stronger (*B* = 2.7, *p* = .003 and *B* = 3.9, *p* = .001, respectively).

Logistic regression was performed to examine a possible association between anaemia and use of HC. In 38-year-olds, a significant decrease in risk of having anaemia was seen among the women who used HC compared to women who did not (OR 0.35, 95% CI 0.15–0.81). When adjusting for the previously mentioned covariates, the odds were further decreased (OR 0.32, 95% CI 0.13–0.75). In 50-year-old women, no significant association was seen between use of HC and risk of having anaemia.

## Discussion

### Principle findings

A significant U-formed non-linear trend in Hb was found in both 38- and 50-year-old women between the examination years 1968–1969 and 2016–2017, with highest levels in the first and the last examination years and lowest in 1992–1993. Although it is not possible to draw any certain conclusions about the reasons for these changes, several lifestyle factors can be assumed to have contributed, not least use of HC, which in this study was shown to be significantly associated with the level of Hb in both 38- and 50-year-old women. The use of HC was, in both age groups, found to be relatively low in 1992–1993 compared to the other examination years, which coincide with the clear decline in Hb for both 38- and 50-year-olds at this point. In addition to the factors shown to be associated with Hb in this present study, also other lifestyle factors have changed, including for example the ages of women when they give birth. Since 1968, there has been a decrease in teenage mothers, and a simultaneous increase in older mothers [27]. Also the number of children that women give birth to has altered over the years, with a peak around the year 1990 [28]. However, it is difficult to determine whether these changes have influenced the later cohorts’ Hb or not. According to WHO, the global anaemia prevalence in women of reproductive age in 2019 was 29.9% and 36.5% in pregnant women. Corresponding numbers for Swedish women were 13.6% (increased from 12.6% in 2000) in women of reproductive age and 18.0% (18.7% in 2000) in pregnant women. The global prevalence of anaemia in women of reproductive age has been stable since 2000 and simultaneously decreased slightly in pregnant women [29]. It is important for caregivers in primary health care meeting pregnant women to be aware of their risk of anaemia and its association with preterm delivery and low birth-weight infants [30]. Anaemia is not only dangerous for the baby, it can also lead to impaired quality of life for the women, and if it remains untreated, it can have serious health effects [1,4]. Specific groups of women, for example, women with severe mental illnesses and complex health needs, and who rely on caregivers in primary health care, are important to recognise and improve reproductive and prenatal planning [31].

Smoking, which is associated with a higher Hb in both previous research and the present study, was more common in 38-year-old women in 1968–1969 than in later examination years [18], possibly contributing to the higher Hb in this group in the first examination year. HC were more common in women in the two latest examinations (2004–2005 and 2016–2017) and might therefore have contributed to the again higher Hb levels in these years. Women using HC were in this study found to have a 2.4–3.0 g/L higher mean Hb level, in line with the result of a lower risk of anaemia for the 38-year-old women using HC.

The decreased risk of anaemia in HC users was not found in the 50-year-old women, possibly since menopausal status has not been considered in the comparison between the two groups. Even though menopausal status in a recent study has been shown to be difficult to investigate in later born 50-year-old women [21], some of the 50-year-old women in the present study could be expected to experience a decrease in menstrual blood loss at the time of the examinations due to menopause or perimenopause. Since menopause could be expected to have the same effect on menstrual bleedings and anaemia risk as HC, albeit risks may be partly masked in the HC user group, it is possible that the lack of a difference between the groups with and without HC in the 50-year-olds was due to this circumstance.

Apart from the covariates smoking and HC, BMI also had a clear significant association with a higher Hb, a result also shown by Skjelbakken et al. [32], while consuming wine showed a somewhat weaker but still significant association with higher Hb in the 50-year-old women and none in the 38-year-olds. The level of mean Hb in 50-year-old women consuming wine compared to the ones not consuming wine was almost one unit (g/L) higher, and a corresponding higher level was also found in the 50-year-old women consuming wine regularly compared to those only consuming it sometimes. This is partially consistent with the findings of another Scandinavian study where alcohol consumers were found to have a higher Hb, with the most prominent effect shown in women [16]. However, in that study, a significant effect on Hb was only seen when drinking exceeded 14 drinks/week (∼170 g ethanol/week) in men and 7 drinks/week (∼85 g ethanol/week) in women [16].

### Strengths and weaknesses

The most prominent strength of this study is the longitudinal design, where new cohorts have been included during the long follow-up time. The design ensures that the questionnaires have been consistent and the examinations performed in the same way throughout the years. The representative sample of this study can also be expected to reflect the levels of Hb and the use of HC in the general population of Swedish women and not a patient derived data register.

Another strength is the meticulous mapping of hormonal medication and menstrual status continuously undertaken in the Population Study of Women in Gothenburg from the very start in 1968.

One of the limitations of this study is that the participation rate of the population study of women in Gothenburg has declined since the start of the study in 1968–1969, from about 90% to about 70% today. However, since 2004–2005, the participation rate has stabilised and can be considered as acceptable for population studies of today. Another limitation is that we only have used the level of Hb and no other tests commonly used in connection with anaemia. Haemoglobin was the only test that was analysed throughout all the examinations.

Another weakness of this study is that women with pronounced difficulties in speaking and understanding Swedish were excluded, which could possibly lead to a weaker representativeness among immigrants [18]. In the latest examination year of 2016–2017, the excluded women were in total 14 (2%), thus still a small share of the total number of participants.

### Findings in relation to other studies

In this study, a significant U-formed trend in Hb was found in 38- as well as 50-year-old women, with highest levels in the first and the last examination. A systematic analysis of population-based data between 1995 and 2011 showed a similar development with increasing Hb after the 1990s, but only for countries not classified as high-income countries. For high-income regions, the same analysis showed little to no difference in Hb levels in women aged 15–49 years during the same time. However, this was partly because these regions were the ones with the least data [8].

The mean Hb level in 38-year-old women was in our study found to be lower than in 50-year-old women, which is consistent with previous results [33]. Since at least some of the 50-year-old women are likely to be postmenopausal, this result can also be considered to be in line with the findings from another population-based study, where Swedish premenopausal women were found to have a lower mean Hb than postmenopausal women [7]. However, ages at inclusion in that study were 44–73 years, thus mostly considerably older than our population.

With regard to our finding that 38-year-old women using HCs had a lower risk of having anaemia, a similar result was found in another study where hormonal and copper devices were compared [15]. In that study, a higher Hb was found in the users of hormonal intrauterine devices a couple of years after insertion of the device [15]. In the population study of women in Gothenburg, a large increase in the use of hormonal intrauterine devices after 1992–1993 has been shown [21], which is likely to have contributed to the decreased prevalence of anaemia seen during the same period of time. However, in the present study, the specific type of HC that the women were using was not considered, since the effect with less menstrual bleedings is described also for oral HCs. Also, subgrouping of different HCs would have made analyses of a possible association with Hb more difficult to perform.

### Meaning of the study, possible mechanisms and implications for clinicians

This study adds to the knowledge about trends in Hb during the last 50 years in Swedish women. Low Hb is common in young women and may continue as they get older, if not treated by their GPs. Identifying anaemia in women of reproductive age is important in order to maintain well-being and prevent future poor health in the form of pregnancy complications and other serious health effects [1,2,4].

Further, the results of this study implicate a possible benefit with use of HC in premenopausal women apart from the contraception itself, since a clearly decreased prevalence of anaemia was found among HC users. This effect could be considered by clinicians in the consultation of younger middle-aged women.

This study shows a relatively large variance in Hb in Swedish women since 1968–1969, results that in some aspects might differ from previous research. Further studies are needed for a more complete picture of trends in Hb and the underlying reasons behind them.

## Appendix Table A. Means and standard deviations in Hb in different examination years

**Table:**

	38-years-old		50-years-old	
Examination year	*n*	Mean (SD)	*n*	Mean (SD)
1968	372	135.18 (10.26)	398	137.24 (11.03)
1980	122	130.58 (9.64)	355	133.21 (11.21)
1992	69	127.72 (12.89)	99	129.25 (10.18)
2004	207	130.66 (10.59)	293	133.15 (11.57)
2016	263	133.11 (10.15)	310	135.76 (9.44)
Total	1033	132.71 (10.66)	1455	134.58 (11.04)
